# Cerebral blood flow based on 3D-ASL technology in the early detection of mild cognitive impairment in type 2 diabetic patients: a pilot study

**DOI:** 10.3389/fendo.2025.1576998

**Published:** 2025-05-14

**Authors:** Tingzhou Hou, Yijie Wang, Hanwen Xu, Zhenyu Shu, Xiaodong He, Xiaohong Wu

**Affiliations:** ^1^ The Second School of Clinical Medicine, Zhejiang Chinese Medical University, Hangzhou, Zhejiang, China; ^2^ Geriatric Medicine Center, Key Laboratory of Endocrine Gland Diseases of Zhejiang Province, Department of Endocrinology, Zhejiang Provincial People’s Hospital (Affiliated People’s Hospital, Hangzhou Medical College), Hangzhou, Zhejiang, China; ^3^ Department of Endocrinology, Jiashan First People’s Hospital, Jia Xing, China; ^4^ Department of Radiology, Center for Rehabilitation Medicine, Zhejiang Provincial People's Hospital (Affiliated People's Hospital, Hangzhou Medical College), Hangzhou, Zhejiang, China

**Keywords:** type 2 diabetes mellitus, mild cognitive impairment, white matter hyperintensities, cerebral blood flow, arterial spin labeling

## Abstract

**Context:**

Type 2 diabetes mellitus (T2DM) significantly increases the risk of mild cognitive impairment (MCI), and earlier recognition is crucial for timely intervention and improving patients’ quality of life.

**Objective:**

The aim of this study was to characterize changes in cerebral white matter hyperintensities (WMH) and cerebral blood flow (CBF) based on 3D-arterial spin labeling (3D-ASL) measurements in T2DM with MCI, and to assess their potential as markers for early prediction of MCI.

**Methods:**

This study included 30 T2DM patients stratified into T2DM-MCI and T2DM-nMCI groups using MMSE/MoCA. MRI assessed cerebral WMH volume (T2-FLAIR) and regional CBF (3D-ASL) in temporal, parietal, occipital, and hippocampal areas. Group differences in WMH/CBF were analyzed, ROC evaluated CBF’s diagnostic efficacy for MCI, and CBF-cognition correlations were assessed via Spearman’s analysis.

**Results:**

Cranial MRI analysis shows that there were no significant differences between the two groups in terms of total cerebral WMH volume and regional volume (P>0.05). CBF in the temporal, parietal, occipital, and hippocampal regions was significantly lower in the T2DM-MCI group than in the T2DM-nMCI group (P<0.05). ROC analysis revealed that CBF in the hippocampus had the highest diagnostic efficacy (AUC = 0.813, optimal cutoff value of 41.165 ml/(100 g·min), sensitivity 73.3%, specificity 80.0%). Spearman’s correlation analysis showed that CBF in the temporal, parietal, occipital, and hippocampal regions was significantly and positively correlated with MoCA scores (P < 0.05), with temporal and parietal CBF also significantly and positively correlated with MMSE scores (P < 0.05).

**Conclusions:**

CBF, based on 3D-ASL measurements, especially in the hippocampus, is a promising functional marker for identifying MCI in patients with T2DM.

## Introduction

Type 2 diabetes mellitus (T2DM) is a metabolic disorder characterized by chronic hyperglycemia, which is not only strongly associated with traditional complications such as cardiovascular disease and diabetic retinopathy, but also significantly increases the risk of cognitive dysfunction (1). Mild cognitive impairment (MCI) (2, 3), characterized by a subtle decline in memory, attention, language, or executive functioning, while the ability to function in daily life remains essentially intact, is of particular concern in these patients. Early identification of MCI in individuals with T2DM is crucial for timely intervention and improving quality of life (4). Although the Brief Mini-Mental Status Examination (MMSE) and Montreal Cognitive Assessment (MoCA) (5) are widely used for assessing cognitive function (6), being behavioral scales, they are limited in their ability to detect microscopic changes in brain structure and function.

Imaging techniques are valuable for revealing brain changes associated with T2DM. Cerebral white matter hyperintensity (WHM), a structural marker of cerebral small vessel disease, has been shown to be associated with cognitive impairment (7, 8). However, its formation is usually a chronic process, with low sensitivity for early diagnosis. In contrast, cerebral blood flow (CBF) measurement based on 3D-arterial spin labeling (3D-ASL) technology is non-invasive, sensitive, and capable of reflecting functional changes in the brain (9), particularly in brain regions closely related to higher cognitive functions, such as the temporal lobe, parietal lobe, and hippocampus (10–12). While the importance of cerebral WHM and CBF in brain diseases has been studied to some extent, there is still a lack of systematic analysis and discussion on the characteristics of these imaging biomarkers and their clinical significance in different cognitive states in patients with T2DM.

The aim of this study was to characterize the changes in cerebral WMH burden and CBF distribution, measured by the 3D-ASL technique, in T2DM patients across different cognitive states, and to assess the value of these imaging features as potential markers for predicting MCI. Additionally, the correlation of CBF with MMSE and MoCA scores was analyzed to reveal its clinical significance in the early identification and intervention of MCI associated with T2DM.

## Methods

### Research design and study subjects

This was a single-center, prospective study that included 30 patients with T2DM, who were seen between April 2022 and December 2023 at Zhejiang Provincial People’s Hospital. All study participants completed the Brief MMSE and the MoCA to assess cognitive function and underwent cranial magnetic resonance imaging (MRI). Based on the Brief MMSE and MoCA scoring criteria, the study subjects were divided into two groups: the T2DM with mild cognitive impairment group (T2DM-MCI group): MMSE < 26 points or MoCA < 26 points; and the T2DM without mild cognitive impairment group (T2DM-nMCI group): MMSE ≥ 26 points and MoCA ≥ 26 points. Patients in the T2DM-MCI group were recruited first. To minimize the influence of demographic variables such as age, gender, and education level on the study results, patients in the T2DM-nMCI group were recruited and matched based on the key demographic characteristics of the T2DM-MCI group. Fifteen patients were finally included in each group. Exclusion criteria included: non-T2DM patients, patients with acute complications of T2DM, patients with previous organic brain diseases, patients with severe psychiatric disorders that could affect the assessment of cognitive function, patients with moderate to severe cognitive impairment, and patients with contraindications to or who refused to undergo MRI. The study strictly adhered to the ethical principles of the Declaration of Helsinki and was approved by the Ethics Committee of Zhejiang Provincial People’s Hospital (QT2024229).

### General clinical information and laboratory tests

General clinical information was collected, including gender, age, education level, smoking history, alcohol consumption, and family history, among others. Screening for diabetic retinopathy was performed by the same experienced ophthalmologist, who independently interpreted the results. Evaluation of diabetic peripheral neuropathy was carried out by the same physician using a uniform, standardized examination procedure. This included tests for vibratory sensation, the 10g nylon wire test, ankle reflexes, temperature sensation, and pinprick nociception. Diagnosis was based on the Chinese Guidelines for the Prevention and Control of T2DM, and results were recorded in dichotomous form (“yes” or “no”). After fasting for more than 8 hours, patients provided blood samples from their veins for laboratory tests early the next morning. All laboratory parameters (including biochemical and diabetes-related indicators) in this study were measured using a uniform testing method to ensure the accuracy and consistency of the data.

### Cognitive function assessment

Cognitive function was assessed by the same systematically trained clinician in all study subjects, and the assessment process followed a strictly standardized procedure to avoid interference or bias. Cognitive assessment tools included the MMSE and the MoCA. The MMSE: a total score of 30, with a score of ≥27 considered normal. MoCA: a total score of 30, with high diagnostic sensitivity and specificity for MCI, and a score of ≥26 considered normal. Diagnostic criteria for MCI: according to the 2018 Chinese Guidelines for the Diagnosis and Treatment of Dementia and Cognitive Impairment, the diagnosis of MCI requires the following conditions: the patient or an informed person subjectively reports a decline in cognitive function, with a scale suggestive of cognitive impairment: a MoCA score of <26 or an MMSE score of 21–26 points, the ability to perform daily activities independently without dependence on others, and the diagnostic criteria for dementia have not yet been met (e.g., MoCA score >18). MRI scanning and data processing.

### MRI scanning and data processing

MRI scanning was performed using a 3.0 Tesla MRI scanner (Discovery MR 750, GE Healthcare). During the scan, subjects were positioned supine with their heads immobilized using cotton pads and wearing noise-reducing earplugs to minimize acoustic interference. Conventional sequence scans, including T1WI, T2WI, DWI, and FLAIR sequences, were initially performed for WMH grading, volume measurements, and exclusion of significant brain lesions. Subsequently, a research sequence 3D-ASL scan was conducted.

### WMH division and volume calculation

The T2-FLAIR-weighted images were imported into the MATLAB platform, and the WMH was automatically segmented using the Lesion Prediction Algorithm (LPA) in the SPM12 toolbox. The WMH volume in 1 mm³ voxel space was calculated for each MRI slice. Two experienced radiologists independently assessed the segmentation results, and poor-quality segmented images were manually corrected and used for analysis. WMH volumes included the following nine brain regions: deep white matter, corpus callosum, frontal lobe, temporal lobe, parietal lobe, occipital lobe, insula, brainstem, and cerebellum. All WMH volumes were calculated in milliliters (mL) and normalized by the ratio to intracranial volume to eliminate the effect of cranial size differences.

### CBF value calculation

3D-ASL sequences were processed in a GE MRI ADW4.6 post-processing workstation using Functool software. Poor-quality images were rejected after a thorough review, and thresholds were adjusted to ensure that the calculation covered the entire brain tissue. The final pseudo-colored CBF images were generated. Measurements were repeated three times within each region of interest (ROI, area 20 mm²), and CBF values were recorded after averaging.

### Statistical analysis

All data analysis was conducted using SPSS 24.0 statistical software. Normally distributed data are expressed as mean ± standard deviation ( $\bar{\text{x}}$ ± s); comparisons between two groups that conformed to a normal distribution with homogeneous variance were performed using the independent samples t-test. The modified t-test was used when the variance was not homogeneous. Measurement data that were not normally distributed are expressed as median and interquartile range M (Q1, Q3), with comparisons between groups made using the rank-sum test. Statistical data were expressed as the number of cases and composition ratio (%), and the chi-square test was used to analyze the differences between groups. The diagnostic value of CBF in predicting MCI in patients with T2DM was analyzed using ROC curves, and the calculated indexes included the area under the curve (AUC), cut-off value, sensitivity, specificity, and Youden’s index. Spearman’s correlation analysis was used to assess the correlation between the CBF values in different brain regions and the MMSE and MoCA scores in the T2DM-MCI and T2DM-nMCI groups. The difference was considered statistically significant with P < 0.05.

## Results

### Baseline characteristics between the T2DM-MCI and T2DM-nMCI groups

There was no statistically significant difference between the T2DM-MCI and T2DM-nMCI Groups in terms of age, gender, years of education, smoking history, alcohol consumption, and baseline characteristics such as diabetes duration (P > 0.05). Additionally, there was no statistically significant difference between the two groups in terms of biochemical indicators (e.g., glycosylated hemoglobin, blood lipids, renal function, etc.) and comorbidities (e.g., diabetic retinopathy, peripheral neuropathy, etc.) (P > 0.05) (**Table 1**).

**Table 1:** The baseline characteristics of the T2DM-MCI and T2DM -nMCI groups.

Variable	T2DM-MCI (n=15)	T2DM-nMCI (n=15)	t or x2 or z	P
Males	12 (80.0%)	13 (86.7%)	0.240	0.624
Age, yr	62.0 (50.0,65.0)	58.0 (43.0,63.0)	-1.206	0.228
Less than or equal to 6 years of education	6 (40.0%)	2 (13.3%)	3.277	0.351
Smoking	7 (46.7%)	8 (53.3%)	0.133	0.715
Drinking	4 (26.7%)	8 (53.3%)	2.222	0.136
Family history of diabetes	3 (20.0%)	6 (40.0%)	1.429	0.232
History of hypertension	8 (53.3%)	3 (20.0%)	3.589	0.058
The course of diabetes, yr	10.0 (7.0,13.0)	2.0 (1.0,10.0)	-1.954	0.056
Systolic pressure, mmHg	138.07 ± 14.32	130.00 ± 12.82	-1.625	0.116
Diastolic pressure, mmHg	82.33 ± 11.13	76.00 ± 9.57	-1.671	0.106
Body mass index, kg/m2	23.85 ± 5.00	22.54 ± 3.27	-0.851	0.403
Alanine aminotransferase, U/L	19.00 ± 8.64	26.40 ± 14.25	1.720	0.099
Aspartate aminotransferase, U/L	21.13 ± 8.47	23.38 ± 7.21	0.760	0.454
Blood urea nitrogen, mmol/L	5.43 ± 1.59	5.86 ± 1.31	0.806	0.427
Serum creatinine, µmol/L	73.3 (65.0,88.5)	74.1 (64.5,82.8)	-0.104	0.917
Uric acid, µmol/L	355.00 ± 133.49	359.67 ± 102.05	0.108	0.915
Triglyceride, mmol/L	4.40 ± 1.69	4.97 ± 1.05	-0.076	0.278
Total cholesterol, mmol/L	2.39 ± 4.70	2.29 ± 2.02	1.111	0.940
High density lipoprotein cholesterol, mmol/L	1.02 ± 0.26	0.99 ± 0.25	-0.269	0.790
Low density lipoprotein cholesterol, mmol/L	2.44 ± 1.00	2.98 ± 0.85	1.566	0.129
Estimated glomerular filtration rate, mL/min/1.73 m^2^	92.39 (80.98,105.34)	93.48 (85.08,108.28)	0.678	0.576
Ratio of microalbumin to creatinine, mg/g	73.77 (27.76,535.00)	17.76 (13.68,93.98)	-1.901	0.061
Diabetic peripheral neuropathy	9 (60.0%)	6 (40.0%)	1.200	0.273
Diabetic retinopathy	2 (13.3%)	1 (6.7%)	0.370	0.543
Glycosylated hemoglobin A1c, %	9.53 ± 2.50	8.85 ± 2.80	-0.702	0.489
Fasting insulin, µU/ml	5.40 (1.68,15.48)	6.10 (1.89,12.07)	-0.103	0.918
2h postprandial insulin, µU/ml	17.29 (8.48,29.49)	25.63 (5.71,58.12)	-0.732	0.464
Fasting C-peptide, ng/ml	0.91 (0.74,1.40)	1.18 (0.66,2.45)	-0.540	0.589
2h postprandial C-peptide, ng/ml	4.97 ± 5.12	5.76 ± 3.94	0.437	0.667
Fasting blood-glucose, mmol/L	6.00 ± 1.85	6.28 ± 2.55	0.326	0.747
2h postprandial blood Glucose, mmol/L	15.54 ± 4.85	15.87 ± 4.82	0.171	0.918

### WMH volume between T2DM-MCI group and T2DM-nMCI group

From the perspective of brain structure. T2DM-MCI Group and T2DM-nMCI Group of study subjects used MATLAB for segmentation of brain regions affected by WHM and calculation of the volume and number of lesions. The results showed no significant difference (P > 0.05) in the total volume of WMH, the volume of WMH in the nine brain regions, or the number of lesions between the two groups (**Table 2**).

**Table 2:** Comparison of WHM volume in T2DM-MCI and T2DM-nMCI groups.

WMH region	T2DM-MCI (n=15)	T2DM-nMCI (n=15)	Z	P-value
Total WMH	23.15 (0.00, 56.48)	23.15 (0.00, 56.48)	Z=-1.69	0.091
Total number of WMH lesions	18.00 (4.50, 30.00)	2.00 (0.00, 8.00)	Z=-1.86	0.063
Deep white matter	5.09 (0.03, 12.28)	0.07 (0.00, 1.28)	Z=-1.79	0.074
Corpus callosum	0.52 (0.00, 2.51)	0.00 (0.00, 0.31)	Z=-1.51	0.131
Frontal lobe	2.24 (0.00, 14.36)	0.00 (0.00, 0.49)	Z=-1.95	0.052
Temporal lobe	5.71 (0.04, 116.80)	1.01 (0.00, 4.73)	Z=-1.35	0.176
Occipital lobe	2.16 (0.13, 15.63)	0.31 (0.00, 2.16)	Z=-1.32	0.187
Parietal lobe	2.91 (0.00, 64.65)	0.08 (0.00, 1.28)	Z=-1.61	0.108
Insula lobe	0.00 (0.00, 0.00)	0.00 (0.00, 0.00)	Z=-0.51	0.609
Cerebellum	0.00 (0.00, 0.00)	0.00 (0.00, 0.00)	Z=-0.93	0.351
Brain stem	0.00 (0.00, 0.00)	0.00 (0.00, 0.00)	Z=-0.93	0.351

### CBF in brain regions between T2DM-MCI group and T2DM-nMCI group and ROC curve analysis

**Figure 1:**
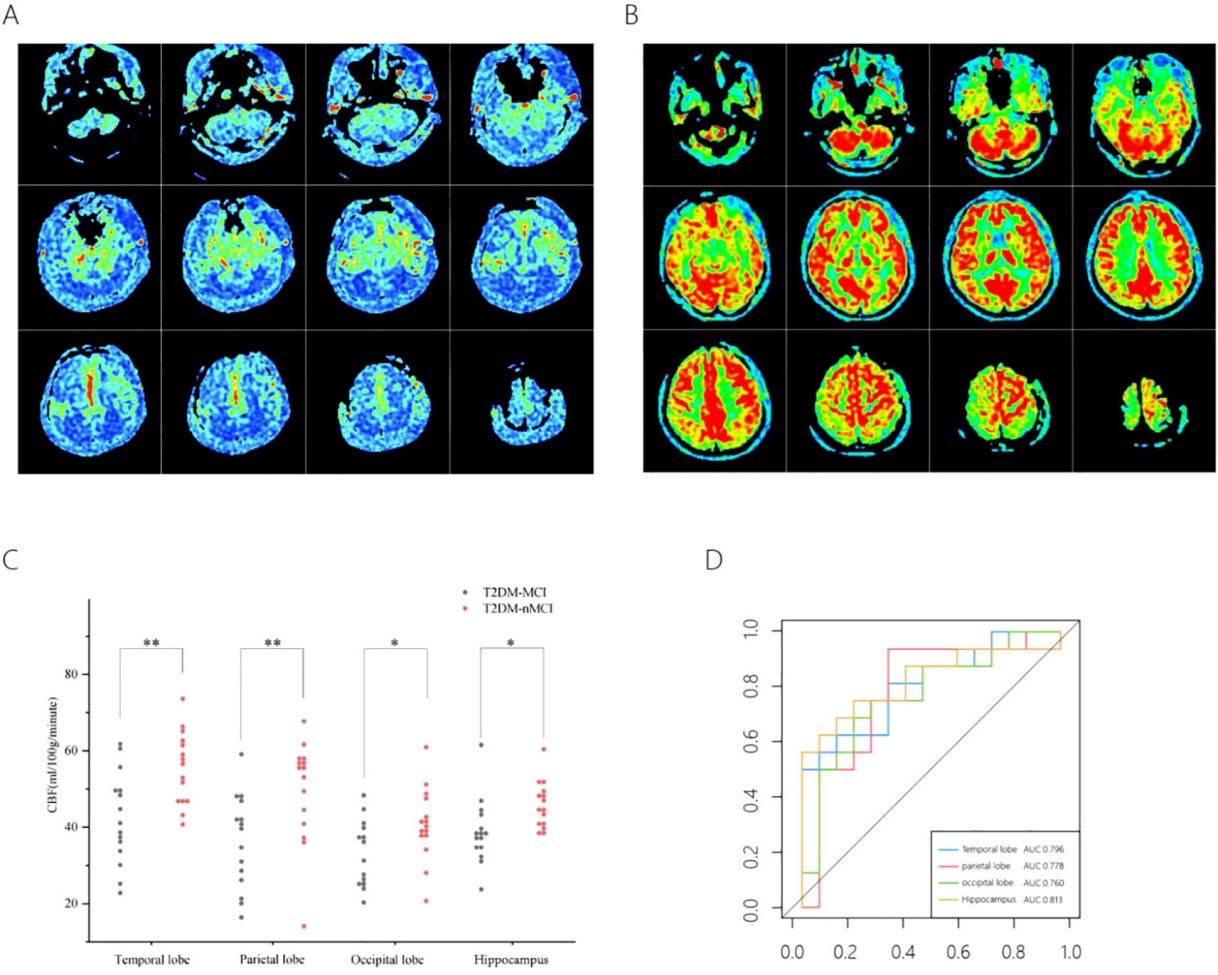
CBF in different brain regions in the T2DM-MCI versus T2DM-nMCI groups. A pseudo-color plot of the CBF images (from red to blue, with red being high perfusion and blue being low perfusion) in 3D-ASL in T2DM-MCI group **(A)**, a pseudo-color plot of the CBF images in 3D-ASL in T2DM-nMCI group **(B)**. The mean CBF scatter plot **(C)**, The ROC curves of the CBF values **(D)**. *P <0.05, **P <0.01 significantly.

The CBF values of the T2DM-MCI group were significantly lower than those of the T2DM-nMCI group overall (**Figures 1A, B**). Further analysis revealed that CBF values were significantly lower in the T2DM-MCI group compared to the T2DM-nMCI group in all four regions: temporal lobe, parietal lobe, occipital lobe, and hippocampus (**Supplementary Table 1**) (**Figure 1C**). ROC curve analysis showed that CBF values in the temporal lobe, parietal lobe, occipital lobe, and hippocampus all had high diagnostic efficacy for T2DM-MCI, with the hippocampus demonstrating the highest diagnostic efficacy (**Supplementary Table 2**) (**Figure 1D**).

### Correlation analysis between CBF values in brain regions and cognitive scores

**Figure 2:**
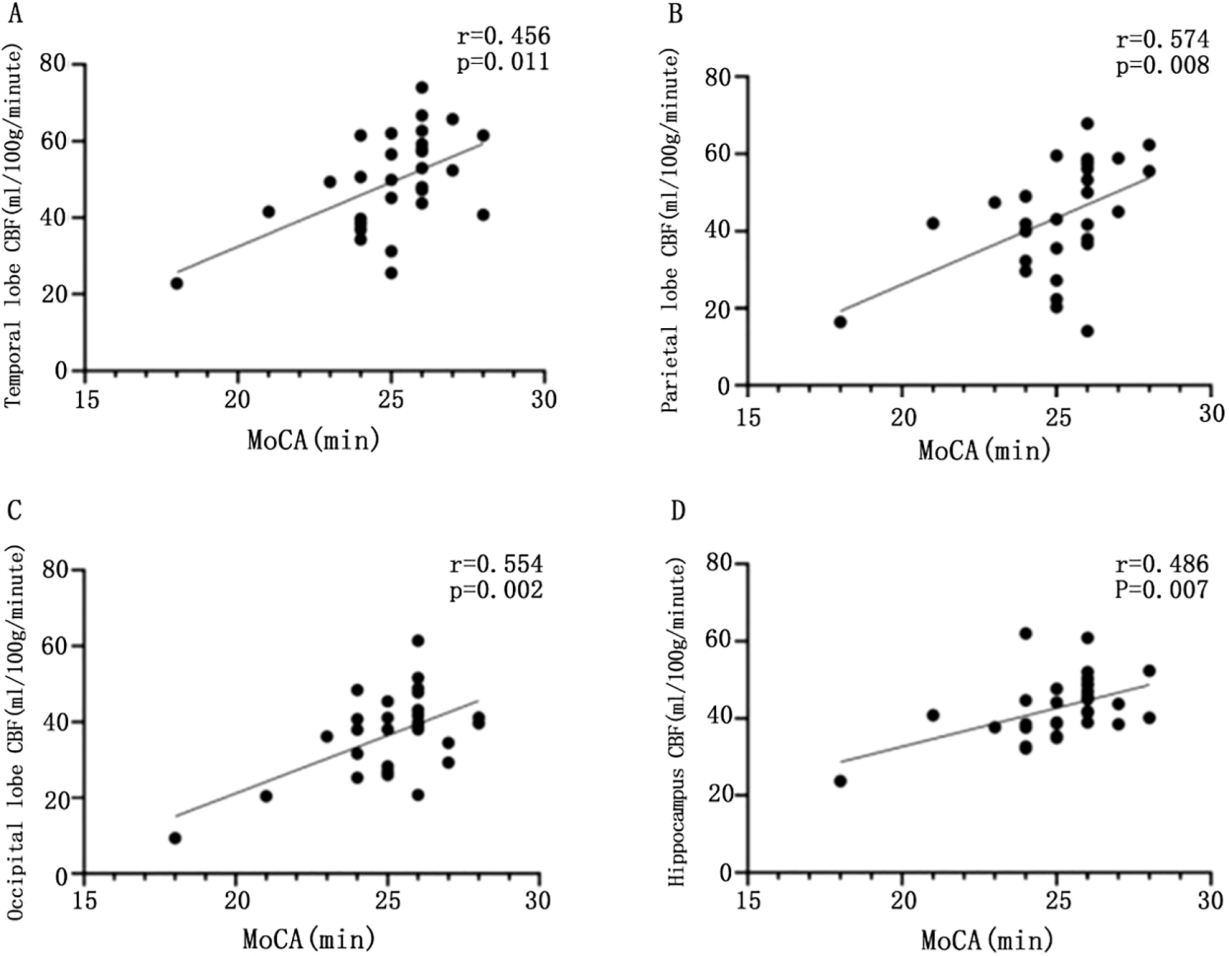
Scatter plot of the correlation between CBF values and MoCA scores in different brain regions. Scatter plot of correlation between CBF and MoCA in temporal lobe of patients with T2DM **(A)**; scatter plot of correlation between parietal CBF and MoCA in patients with T2DM **(B)**; scatter plot of correlation between CBF and MoCA in patients with T2DM **(C)**; scatter plot of correlation between CBF and MoCA in patients with T2DM **(D)**, with statistically significant difference at P <0.05.

Based on the above conclusions, we further explore the relationship between CBF and cognition. We found that CBF values in the temporal (r = 0.529, P = 0.003), parietal (r = 0.574, P = 0.008), occipital (r = 0.554, P = 0.002), and hippocampal (r = 0.486, P = 0.007) regions were significantly and positively correlated with MoCA scores (**Figure 2**).

**Figure 3:**
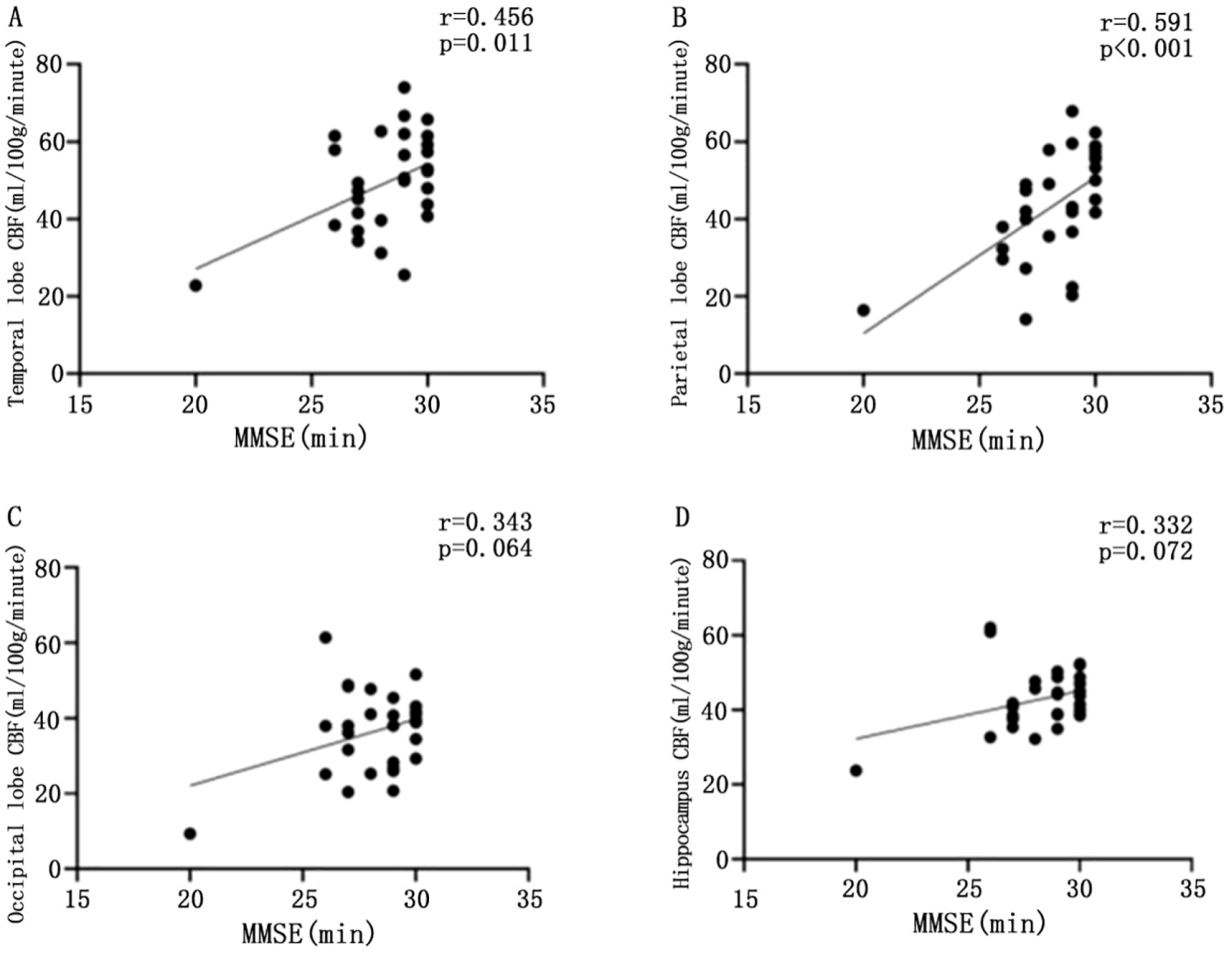
Scatter plot of the correlation between CBF values and MMSE scores in different brain regions. Scatter plot of correlation between CBF and MMSE in temporal lobe of patients with T2DM **(A)**; scatter plot of correlation between parietal CBF and MMSE in patients with T2DM **(B)**; scatter plot of correlation between CBF and MMSE in patients with T2DM **(C)**; scatter plot of correlation between CBF and MMSE in patients with T2DM **(D)**, with statistically significant difference at P <0.05.

CBF values in the temporal lobe (r = 0.456, P = 0.011) and parietal lobe (r = 0.591, P < 0.001) were significantly and positively correlated with MMSE scores. However, CBF values in the occipital lobe (r = 0.343, P = 0.064) and hippocampus (r = 0.332, P = 0.072) did not show a significant correlation with MMSE scores (P > 0.05) (**Figure 3**).

## Discussion

In this study, we investigated the differential performance of cerebral WMH and CBF in patients with T2DM with MCI. Our study found that the T2DM-MCI group and the T2DM-nMCI group did not show significant differences in total cerebral WMH volume and regional brain volume. And CBF was significantly lower in the temporal, parietal, occipital, and hippocampal regions of the T2DM-MCI group. These regions are closely related to higher cognitive functions such as memory, attention, and executive function. ROC analysis further showed that CBF, particularly in the hippocampal region, had high diagnostic efficacy. The correlation between CBF and MoCA scores, as well as MMSE scores, further supports its significant value in assessing cognitive function. These results suggest that functional alterations in CBF may precede the appearance of structural damage, such as cerebral WMH, and may become an important imaging marker for the early prediction of MCI in patients with T2DM.

Cerebral WMH is an important structural marker of brain aging and cerebrovascular disease, and has been widely recognized as being closely associated with cognitive dysfunction (8). In patients with T2DM, chronic hyperglycemia may accelerate cerebral WMH through mechanisms such as microangiopathy, chronic inflammation, and oxidative stress (13). Previous studies have shown that increased high signal burden in the brain white matter of T2DM patients is closely associated with cognitive decline (14, 15), while others have shown no significant correlation between the two (16). This study also did not find significant differences in the total volume of cerebral WMH and regional brain volume between the T2DM-MCI and T2DM-nMCI groups. This may be related to the fact that the formation of cerebral WMH is a slow, cumulative process, which occurs more in the middle to late stages of cognitive dysfunction rather than in the early stages. Thus, cerebral WMH may not be a sensitive early predictor of MCI associated with T2DM. Nonetheless, cerebral WMH are still valuable in reflecting the state of cerebrovascular health.

Alterations in CBF appear earlier than structural indicators, such as cerebral WMH, suggesting early impairment of local neurometabolic and microvascular function. This makes 3D-ASL uniquely suited for the early identification of cognitive impairment. 3D-ASL utilizes radio frequency pulses to magnetically label arterial blood protons, integrates 3D imaging with background suppression, and enables non-invasive, whole-brain quantitative blood flow measurement. This technology has the significant advantage of avoiding radiation exposure compared to PET-CT and SPECT, making it more suitable for repeat testing and dynamic observation. The results of relevant studies show that there is no significant difference between CBF maps based on ASL and SPECT, and ASL is more sensitive to hemodynamic changes, which can be considered to be at a higher clinical level (17).

In this study, we utilized 3D-ASL technology to quantify CBF distribution in a noninvasive manner and found that total CBF and CBF in the temporal, parietal, occipital, and hippocampal regions were significantly lower in T2DM-MCI patients than in T2DM-nMCI patients. It has been shown that CBF values assessed by ASL correlate with MCI (17, 18) and AD (19). Multiple interconnected pathways through which CBF alterations may drive cognitive decline in T2DM: 1) Neurovascular coupling disruption: The brain’s ability to dynamically regulate CBF in response to neuronal activity (neurovascular coupling) is impaired in chronic hyperglycemia (20). Our observed hippocampal CBF reductions may reflect this decoupling, particularly in metabolically demanding regions. 2) Blood-brain barrier (BBB) breakdown: Altered CBF is also a significant mechanism contributing to cognitive decline in patients with T2DM. Microvascular and macrovascular complications in diabetic patients lead to reduced blood flow to the brain, thereby affecting the energy supply to brain tissue and impairing neural function. In a hyperglycemic state, the accumulation of advanced glycation end products damages vascular endothelial cells and neuronal cells, further exacerbating neuropathy in diabetic patients (21). And the present study found that reduced total cerebral CBF was associated with mild cognitive dysfunction, further demonstrating that CBF may be an important biomarker for cognitive dysfunction. Moreover, the functional changes identified in our study are closely related to higher cognitive functions, with the temporal lobe and hippocampus primarily responsible for memory encoding and retrieval, the parietal lobe involved in attention and executive functions, and the occipital lobe involved in processing visual information. Decreased CBF suggests impaired localized neurometabolic and microvascular function. Reduced CBF in the temporal, parietal, occipital, and hippocampal regions have been widely reported in Alzheimer’s disease (22, 23) and in patients with MCI (10–12), with decreased CBF in these regions recognized as an important imaging hallmark for individuals at high risk for Alzheimer’s disease. The diagnostic value of temporal lobe and hippocampal CBF has also been validated in patients with MCI in T2DM (7, 24), which is further supported by our study. And our study evaluated the diagnostic efficacy of CBF for MCI in four regions for the first time. Among these, CBF in the hippocampus is not only an important marker of MCI in T2DM (25, 26), but is also associated with memory and executive function performance (27). It may also serve as a target for early intervention. Our study further suggests that CBF in the hippocampus has the best diagnostic efficacy for MCI.

Traditional cognitive assessment scales have significant advantages in screening for cognitive dysfunction, but as behavioral scales, they are unable to capture microstructural and functional changes in brain tissue, particularly in the early stages of cognitive impairment. CBF measured by 3D-ASL can compensate for this limitation and provide neuroimaging evidence that traditional scales cannot. On the other hand, while previous studies have shown an association between elevated CBF and better cognition and mobility (28–30), our study found significant positive correlations between CBF values in the temporal lobe, parietal lobe, occipital lobe, and hippocampus with MoCA scores in patients with T2DM, and significant correlations between CBF values in the temporal lobe and parietal lobe and MMSE scores in patients with T2DM. The significant link between CBF and MCI was not only emphasized but also focused on different brain regions, further highlighting the importance of a comprehensive assessment that combines advanced imaging techniques with traditional scales, and providing theoretical support for the early identification and intervention of MCI associated with T2DM.

There are some limitations in this study: Firstly, this was a pilot study with small size at a single tertiary center, which may not effectively represent the broader population of T2DM patients, introducing some selection bias. This underscores the need for larger validation cohorts. Secondly, the T2DM-MCI group exhibited longer diabetic duration (median 10 vs. 2 years) approaching statistical significance (P=0.056). Prolonged diabetic duration elevates risk of microvascular and macrovascular complications, potentially impairing CBF and cognition, which might impact our experimental findings. Thirdly, our ROC-derived hippocampal CBF threshold (41.165 ml/100g/min) demonstrated good diagnostic accuracy (AUC=0.813). But the generalizability of this finding as a clinical standard needs to be verified in larger-scale and multi-center study. Finally, this study is cross-sectional, while this study identified alterations in CBF in different brain regions in T2DM patients with MCI, the causal relationship between the onset of MCI and changes in CBF was not explored and future longitudinal studies with a follow-up period are needed to investigate the correlation between CBF reduction and cognitive dysfunction at different stages of disease progression in T2DM patients with MCI.

In conclusion, our study found that CBF based on 3D-ASL measurements might an important functional marker for the early identification of MCI in patients with T2DM, especially in the hippocampal region, which shows high diagnostic efficacy. CBF was significantly correlated with MoCA and MMSE scores, and the use of imaging techniques in conjunction with traditional behavioral scales may positively impact the diagnostic accuracy of MCI.

## Data Availability

The raw data supporting the conclusions of this article will be made available by the authors, without undue reservation.
