# Supplementary material for: Cerebral blood flow based on 3D-ASL technology in the early detection of mild cognitive impairment in type 2 diabetic patients: a pilot study
Source: Front Endocrinol (Lausanne). 2025 May 14;16:1576998. doi: 10.3389/fendo.2025.1576998 (PMC12116335; doi:10.3389/fendo.2025.1576998)
Supplement: Supplementary file 1 [file Table1.docx]

**Supplementary materials**

**Table 1** Comparison of CBF values in different brain regions between the T2DM-MCI group and the T2DM-nMCI group

| **names** | **T2DM-MCI** | **T2DM-nMCI** |
| --- | --- | --- |
| Temporal lobe | 43.04±12.06ml/(100g·min) | 55.88±9.40ml/(100g·min)(P=0.003) |
| Parietal lobe | 37.05±12.22ml/(100g·min) | 50.25±13.44ml/(100g·min)(P=0.009) |
| Occipital lobe | 32.06±10.39ml/(100g·min) | 41.20±9.44ml/(100g·min)(P=0.018) |
| Hippocampus | 39.32±8.52ml/(100g·min) | 46.37±6.06ml/(100g·min)(P=0.015) |

**Table 2** The C-statistic of CBF values in different brain regions for predicting T2DM-MCI

| **names** | **AUC(95CI)** | **Cut off** | **sensitivity** | **specificity** | **Youdun Index S** |
| --- | --- | --- | --- | --- | --- |
| Temporal lobe | 0.796（0.634-0.957） | 51.519 | 0.800 | 0.667 | 0.467 |
| Parietal lobe | 0.778（0.597-0.958） | 49.565 | 0.933 | 0.667 | 0.600 |
| Occipital lobe | 0.76  （0.582-0.938） | 37.980 | 0.667 | 0.800 | 0.467 |
| Hippocampus | 0.813（0.649-0.978） | 41.165 | 0.733 | 0.800 | 0.533 |
